# Supplementary material for: Iterative improvement in the automatic modular design of robot swarms
Source: PeerJ Comput Sci. 2020 Dec 7;6:e322. doi: 10.7717/peerj-cs.322 (PMC7924708; doi:10.7717/peerj-cs.322)
Supplement: Supplemental Information 3 [file peerj-cs-06-322-s003.zip › argos3/doc/api/standalone/a00452_source.html]

ARGoS: plugins/robots/foot-bot/control\_interface/ci\_footbot\_turret\_encoder\_sensor.cpp Source File


- Main Page
- Related Pages
- Namespaces
- Classes
- Files

- File List
- File Members

# plugins/robots/foot-bot/control\_interface/ci\_footbot\_turret\_encoder\_sensor.cpp

Go to the documentation of this file.

```
00001 
00007 #include "ci_footbot_turret_encoder_sensor.h"
00008 
00009 #ifdef ARGOS_WITH_LUA
00010 #include <argos3/core/wrappers/lua/lua_utility.h>
00011 #endif
00012 
00013 namespace argos {
00014    
00015    /****************************************/
00016     /****************************************/
00017    
00018    const CRange<CRadians> CCI_FootBotTurretEncoderSensor::ANGULAR_RANGE(CRadians(-ARGOS_PI), CRadians(ARGOS_PI));
00019    
00020    /****************************************/
00021    /****************************************/
00022    
00023    const CRadians& CCI_FootBotTurretEncoderSensor::GetRotation() const {
00024      return m_cRotation;
00025    }
00026 
00027    /****************************************/
00028    /****************************************/
00029    
00030 #ifdef ARGOS_WITH_LUA
00031    void CCI_FootBotTurretEncoderSensor::CreateLuaState(lua_State* pt_lua_state) {
00032       CLuaUtility::OpenRobotStateTable(pt_lua_state, "turret");
00033       CLuaUtility::AddToTable(pt_lua_state, "rotation",  m_cRotation);
00034       CLuaUtility::CloseRobotStateTable(pt_lua_state);
00035    }
00036 #endif
00037 
00038    /****************************************/
00039    /****************************************/
00040 
00041 #ifdef ARGOS_WITH_LUA
00042    void CCI_FootBotTurretEncoderSensor::ReadingsToLuaState(lua_State* pt_lua_state) {
00043       lua_getfield(pt_lua_state, -1, "turret");
00044       lua_pushnumber(pt_lua_state, m_cRotation.GetValue());
00045       lua_setfield(pt_lua_state, -2, "rotation");
00046       lua_pop(pt_lua_state, 1);
00047    }
00048 #endif
00049 
00050 
00051    /****************************************/
00052    /****************************************/
00053 
00054 }
```

---

Generated on 10 Jul 2018 for ARGoS by 
 1.6.1
